# Total cost of ownership of heat pumps and policy choice: The case of Great Britain

**DOI:** 10.1016/j.isci.2025.111784

**Published:** 2025-01-10

**Authors:** Jan Rosenow, Jacob Barnes, Ray Galvin, Samuel O'Mara, Richard Lowes

**Affiliations:** 1 University of Oxford, Environmental Change Institute, 3 South Parks Road, Oxford OX1 3QY, UK; 2 University of Cambridge, Cambridge Institute for Sustainability Leadership, Entopia Building 1 Regent Street, Cambridge CB2 1GG, UK; 3 Gemserv, 2nd floor, 77 Gracechurch St, London EC3V 0AS, UK; 4 University of Exeter, Energy Policy Group, Penryn Campus, Treliever Road, Penryn, Cornwall TR10 9FE, UK

**Keywords:** Energy resources, Energy policy, Economics

## Abstract

Heat pumps are essential for decarbonizing heating, as shown by numerous studies. Their adoption depends significantly on economic attractiveness. Using Great Britain (GB) as a case study, this paper examines the total cost of ownership (TCO) for heat pumps versus gas boilers. TCO is calculated using official energy statistics, field trial data, and residential energy prices, alongside scenario analyses on business as usual, shifting levies from electricity bills to general taxation or to gas bills. Findings reveal that heat pumps provide cost savings for units performing at an above-average efficiency under standard tariffs but yield significant savings with smart tariffs. Results indicate that a carbon tax on gas, matching electricity permit prices, has limited impact. However, shifting levies from electricity to general taxation significantly enhances TCO compared to gas heating, with even greater incentives when levies are shifted to gas heating.

## Highlights

• With an SCOP of 3.3 today a heat pump reaches TCO parity in the UK
• Shifting levies from electricity to general taxation improves the TCO considerably
• Cost incentives become larger if levies and taxes are also attributed to gas heating
• Higher efficiencies and/or ToU and specific heat pump tariffs lead to large cost savings

## Graphical abstract

**Figure:**
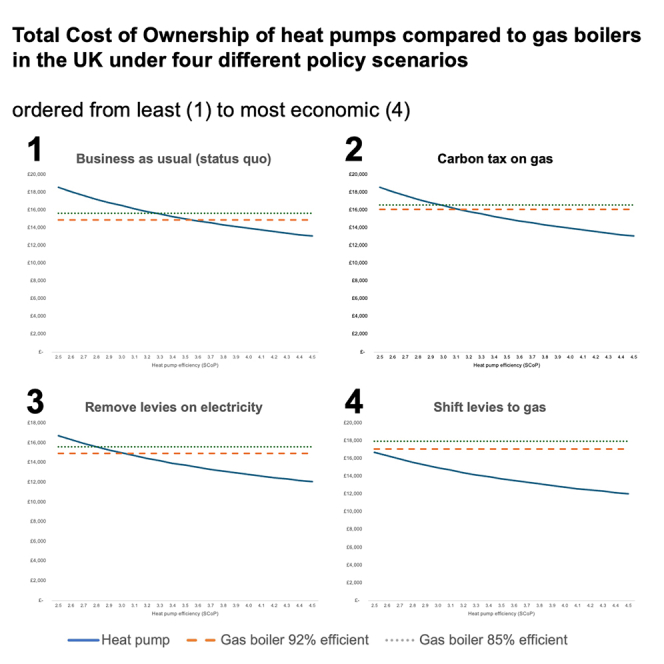

## Introduction

Heat pumps are a mature technology that has achieved dominant market concentrations in a few countries—notably Norway, Sweden, and Finland.^1^ But in many markets, including North America, Europe, and Northern and Eastern Asia where they are expected to play an increasingly central role in the future, heat pumps face a variety of social, economic, and technical barriers.^2^ Overcoming these barriers is a central energy policy challenge around the world.

By 2050, if cost-effective decarbonization is to be achieved, approximately 55% of all buildings are expected to be using a heat pump, equating to about 1.8 billion heat pumps across the global building stock.^3^ In Europe, the European Union (EU) has set a target of installing an additional 30 million hydronic heat pumps across Europe by 2030, increasing the total stock to 60 million installed units.^4^ Meanwhile, the United Kingdom (UK) has set a target of 600k heat pump installations per year by 2028.^5^ Achieving these levels of deployment will require the transformation of contemporary heating systems and will necessitate overcoming a variety of known (and potential some unknown) obstacles.

One of these challenges is the economics of heat pumps compared to fossil fuel heating system and how to make heat pumps economically attractive through consistent price signals that encourage heat pump deployment and use. If overall lifetime costs and running costs of heat pumps remain higher than fossil fuel equivalents, deployment is expected to remain low and emissions reductions targets are unlikely to be met.^6^ In this paper, we review options open to policy makers to make operating a heat pump economically attractive compared to fossil fuel heating systems. We use the UK as a case study as a country that has set ambitious targets yet struggled to deliver significant market growth in the past.

We develop four policy scenarios for rebalancing taxes and levies between electricity and gas and assess their impact on the total cost of ownership (TCO) of a residential air source heat pump compared to a gas boiler, the most common heating system in the UK.^7^ In doing so, we contribute to contemporary debate within the UK and further afield about policy options available to national governments to create consistent price signals that encourage heat pump deployment and use. Most TCO analyses of electrification have been carried out in the transport sector e.g.,^8^ with less work on heating systems that is no longer up to date for the UK.^9^^,^^10^ There is more advanced cost-benefit analysis of heat pumps available for other countries such as the United States.^11^

### Scenarios for rebalancing taxes and levies

The scenarios analyzed in this paper have been presented elsewhere as potential options based on real-world examples,^12^ which also featured in the Mission Zero review ordered by the former UK Prime Minister.^13^ In the following, we briefly outline the core characteristics of each scenario, which will be compared against a business as usual scenario (BAU) in the following analysis. Finally, we set out how expected shifts to dynamic time-of-use pricing also requires consideration when reviewing the economics of heat pumps under different policy scenarios. Our four scenarios are as follows:

(1) Business as usual (BAU)
(2) Shifting levies from electricity to general taxation
(3) Shifting levies from electricity to gas
(4) Applying a carbon tax on gas

Value added tax (VAT) is currently at 5% on electricity and gas for domestic consumers, and VAT could be used as a tool for rebalancing. However, there is no evidence that this is currently being considered for the UK and other countries, so the wider value of VAT modification consideration may be limited.

### Shifting levies from electricity to general taxation

Levies recover costs associated with environmental and social policy objectives via consumer energy bills (Table 1).^12^ The application of levies to consumer energy prices is not new: they have been widely used across Europe since at least the early 1990s.^14^ For a variety of historical reasons such as every household having an electricity meter, levies have been applied to electricity more heavily than gas.^9^^,^^15^^,^^16^

**Table 1:** Levies forecast methodology

Scheme	Forecast methodology
Renewable obligation	The RO scheme is closed and so using the average contract length, the year in which funding requirements for certain capacities of generation could be estimated using historic data on the scheme.^59^
Feed-in tariff	The FiT scheme is closed to new applicants and so a phase out of funding requirements was assumed according to average contract lengths and historical data on Feed-in Tariff contracts.^60^
Contracts for difference	Forecasts for the average weighted strike price and total funded capacities were taken from the Low Carbon Contracts Company.^61^ Using forecasted wholesale electricity prices, the subsidy per unit of generation could be estimated as well as total funding requirements.
Energy Company Obligation	It was assumed that current funding requirements for the core ECO scheme continued at the current rate, according to data on historic ECO scheme costs.^60^ The scheme costs were also adjusted according to the Great British Insulation scheme, with estimated total funding requirements divided equally across the scheme’s lifetime.
Warm Homes Discount	Current funding requirements were assumed to continue, accounting for inflation and historic data on funding requirements.
Green Gas Levy	The scheme was assumed to continue until the financial year 2036/2037 at the current rate.

One option to limit upward price pressures on electricity that encourages the shift away from fossil fuel heating to heat pumps is to remove all levies from electricity to general taxation. This approach has been adopted by Germany with changes having come into force in June 2022. The approach lowers electricity costs proportionate to the amount of levies previously allocated to electricity.^12^

### Shifting levies from electricity to gas

Since most environmental and social levies are attributed to electricity (see for instance^9^), a second means to create more consistent price signals in favor of heat pumps is to shift levies currently allocated to electricity on to gas. One approach could be to immediately allocate all levies to gas that are currently applied to electricity, an approach adopted in our analysis.

Another approach could see levies apportioned according to environmental externalities as suggested by Rosenow et al.^12^ This implies a gradual transfer of levies from electricity to fossil fuels as electricity is progressively decarbonized. Such an approach has been adopted in the Netherlands for energy taxes, where policy makers have increasingly shifted taxes from electricity to gas. The energy tax on gas has experienced a consistent rise, coupled with a reduction in the energy tax on electricity. Tax rates for the typical household consumer have surged by 84% for gas but declined by 25% for electricity from 2013 to 2020.^12^ To offset the impact of high energy taxes, a lump sum tax reduction is provided per household.

Over time, as households move away from gas and adopt heat pumps, fewer and fewer households would have to pay for levies applicable in a given year. This has important equity implications.

### Applying a carbon tax on gas

Environmental taxes have been applied to elements of energy in the UK since the introduction of the European Union’s Emissions Trading Scheme (EU ETS) in 2005. In a “cap and trade” scheme, the EU ETS caps the amount of carbon that can be emitted by large business and creates a market for trading carbon allowances, which have to be surrendered by companies at the end of each year. Following Brexit, the UK Emissions Trading Scheme came into force on 1 January 2021 with many of the features of the EU ETS. The EU and UK ETS place costs on large energy generators, which are passed through to consumers as increased wholesale energy costs. In April 2013, a UK Carbon Floor Price (CFP) on electricity generation was introduced to further support the price of carbon in the UK.

Costs associated with emissions trading and the price floor affect electricity but do not cover gas or other fossil fuels burned in domestic boilers, leading to an obvious carbon tax imbalance.

In the following analysis, a carbon tax of £50/t CO2 is applied to gas. Many other countries in Europe have started applying carbon taxes to fossil heating fuels.^12^ From 2027, the EU will also extend the European Emissions Trading System to buildings, transport, and smaller industrial energy users. No such plans currently exist for the UK.

### (Smart) Time-of-use pricing and heat pump tariffs

ToU tariffs charge a higher or lower price for electricity depending on the extent of demand on the energy grid at any one time. Smart ToU tariffs are enabled by smart meters and vary dynamically according to demand throughout the day, rewarding consumers for using less energy during peak times of demand and using more when there is surplus renewable generation. The overall aim of ToU tariffs is to incentivize consumers to shift their energy use to times when demand is lower, reducing total generation capacity needed and grids stress.^17^ In the UK, ToU tariffs sit alongside smart metering and renewable generation as core elements in the Government’s approach to create smarter, cleaner, more flexible energy systems.^18^^,^^19^

By their nature, smart ToU tariffs are variable and therefore hard to model. In the following analysis, we model results for Octopus Energy’s Agile Tariff. This tariff was introduced to encourage households to shift their energy consumption away from the peak period of 4:00 p.m. to 7:00 p.m., during which the supplier incurs high wholesale prices. By avoiding this peak, consumers contribute to significant savings. When wholesale costs are low or even negative, this allows Octopus Energy to pass on those benefits to customers. The Agile Tariff varies every 30 min with customers receiving the next day’s rates, per 30 min interval, around 4:00 p.m. the day before. In our model, we assume the same relative cost reduction per year as in January to March 2024 averaging £0.16p/kWh (https://agileprices.co.uk/), resulting in a per unit cost reduction of 45% compared to the Ofgem energy price cap during the same period,^20^ a benchmark for retail energy prices in the UK. To what extent time-varying prices will offer similar cost savings in the future is uncertain. For the purpose of this paper we model the TCO assuming a 45% unit cost reduction of future projected time-varying electricity prices. It is uncertain to what extent time-varying tariffs will continue to provide savings of this magnitude and the results need to be treated with caution.

OVO Energy started offering a special heat pump tariff recently (currently limited to the first 100 customers) that offers a flat rate, reduced electricity price for heat pump customers. Their Heat Pump Plus tariff is £0.15/kWh (https://www.ovoenergy.com/heat-pumps), representing a 48% unit price reduction compared to the price cap. Note that the OVO offering is contingent on working with a specific accreditation scheme only covering part of the market and achieving average SCOPs of 4.0.^21^ We calculated TCO under the OVO heat pump tariff, assuming a 48% unit price discount in the future. Whether or not the OVO offering will be available in the future is of course uncertain and needs to be considered when interpreting the results.

In our modeling, we also assess the impact on TCO of those tariffs by applying the average saving made by a standard customer on Octopus’ Agile Tariff and OVO Energy’s flat rate heat pump tariff. Further details are provided below.

## Results

A series of scenarios were modeled, using Equations 1, 2, 3, 4, 5, 6, 7, and 8 in the methodology. These cover business as usual, levies shifted from electricity to the public budget, levies shifted from electricity to gas, and a carbon tax on gas.

### Business as usual

**Figure 1:**
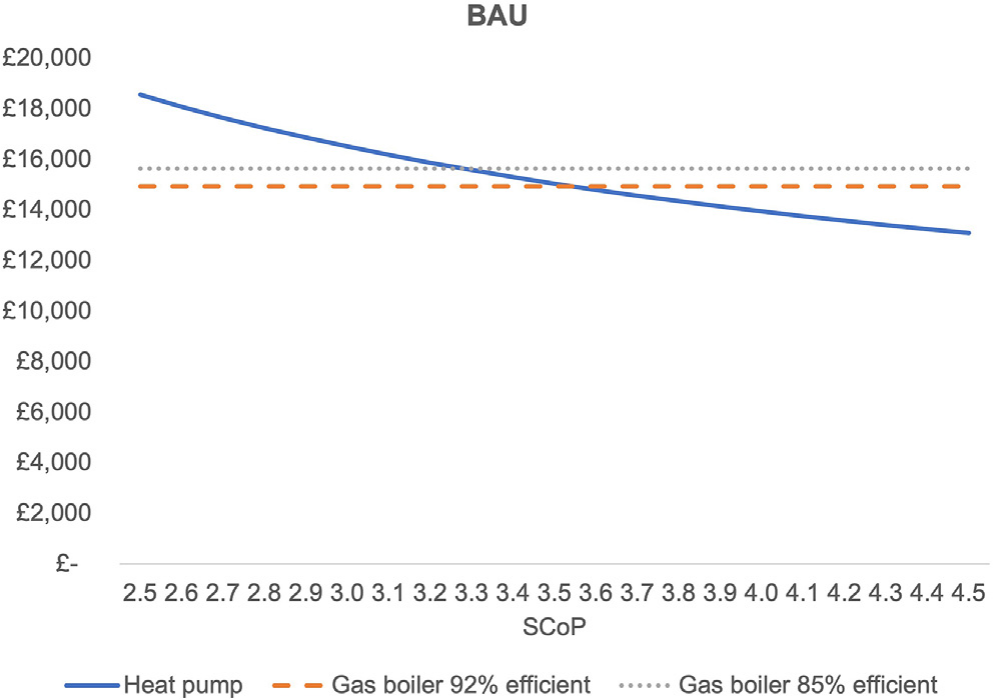
TCO under BAU scenario, for a range of SCOPs Installation cost data for heat pumps have been obtained from MCS.^22^ For gas boiler installation costs, HM Government^23^ data are applied. Average gas demand has been used to derive the average heat demand using Department for Energy Security and Net Zero statistics on household gas use,^24^ energy end-use data tables from the Department for Business, Energy & Industrial Strategy,^25^ and Energy Saving Trust^26^ field data on boiler efficiency. Electricity and gas price data projections have been obtained from DESNZ^27^ Green Book Assumptions. Future standing charges were estimated according to the average historical ratio of unit charges to standing charges from the Ofgem^20^ price cap methodology. A discount rate of 3.5% has been used based on HM Treasury^28^ Green Book guidance used for policy appraisal.

As described earlier, under the BAU scenario we assume no policy changes are introduced (levy reform, carbon tax or else) and prices are forecasted using recent projections based on current market conditions. Figure 1 depicts the TCO following the methodology set out above for a range of SCOPs (2.5–4.5) and gas boilers at two different levels of efficiency (92% and 85%). The results show that cost parity with a gas boiler at 85% efficiency is reached for a heat pump with an SCOP of 3.3 or more.

The Electrification of Heat Trial^29^ showed a median SCOP of 2.9. At an SCOP of 2.9, the TCO of heat pumps exceeds the TCO of gas boilers at 85% efficiency. At an SCOP of 3.3, heat pumps become cheaper than gas boilers from a TCO perspective. Although this is certainly achievable as data show,^21^ this is not currently the situation for the majority of homes where heat pumps have been fitted.

### Shift levies from electricity to general taxation

**Figure 2:**
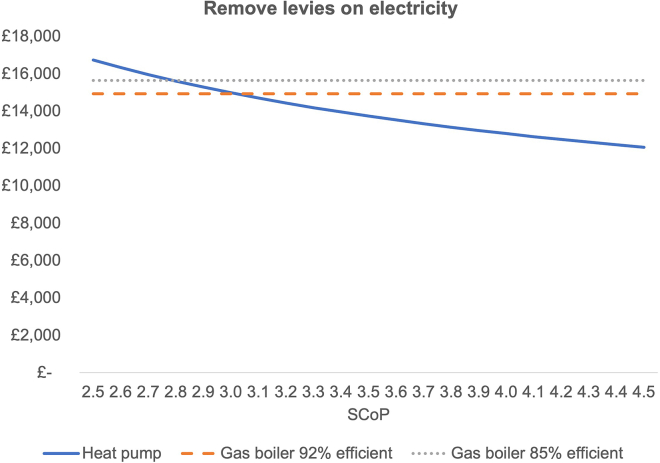
TCO under removing levies scenario The same input data were used as for Figure 1. In addition, data on levies was computed. Energy regulator Ofgem^20^ provides cost data for levies on electricity and gas bills. All levies have been recalculated in £/kWh using the median electricity consumption.^24^ To calculate future levies in £/kWh, the following assumptions have been made: to estimate the future energy levies on consumer bills, complementary data on the current and historic consumption of natural gas and electricity by end use was taken from the Department of Energy Security and Net Zero’s annual Energy Trends data.^30^^,^^31^ This allows for the estimation of current funding pots for relevant schemes funded via levies on domestic consumer bills and is a useful cross reference for future levy forecasts. For each individual scheme funded via levies on consumer bills, a different approach was taken to forecasting the total funding requirement over time. Where appropriate, total funding requirements were scaled down according to projections in future energy usage^32^ and the share of non-domestic consumption. Total funding requirements were then mutualized across the total eligible energy consumption or total eligible customer base according to the current funding structure and data from the National Grid ESO’s Consumer Transformation scenario^33^ on future energy consumption.

If the levies were to be removed from electricity bills and put into the public budget, the TCO of heat pumps would be reduced by £1,642. The recalculated TCO are presented in Figure 2, showing gas boilers under BAU scenario compared to heat pumps with levies removed.

The required SCOP to break even from a TCO perspective is 2.8, less than what has been achieved as a median under the recent trials.^29^ In other words, if an SCOP of 2.9 remains the median of UK, heat pump installs shifting levies to the public budget are sufficient to reach TCO parity between gas boilers and heat pumps. However, this of course relies on the BUS grant to be available in the future.

### Shift levies from electricity to gas

**Figure 3:**
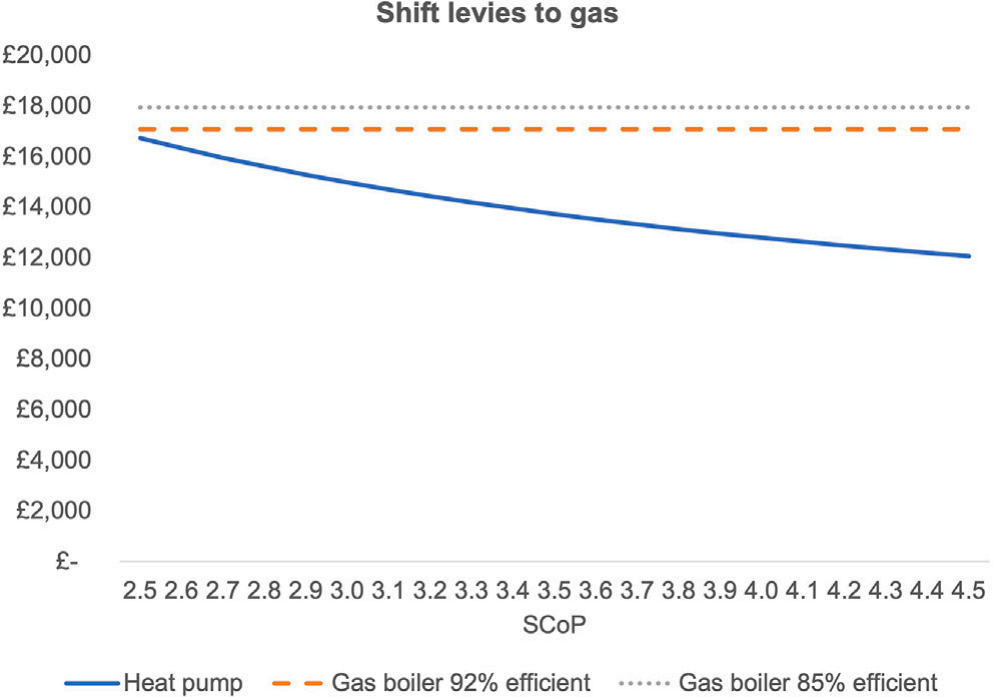
TCO under moving levies to gas scenario, for range of values of SCOPs The same input data as for Figure 2 were used with levies on electricity reattributed to gas unit prices.

By shifting levies from electricity to gas, rather than to the public budget, the impact on TCO would be more substantial, as depicted by Figure 3.

The required SCOP to break even under this scenario is just 2.3 and well below SCOPs from recent field trials.

### Carbon tax on gas

**Figure 4:**
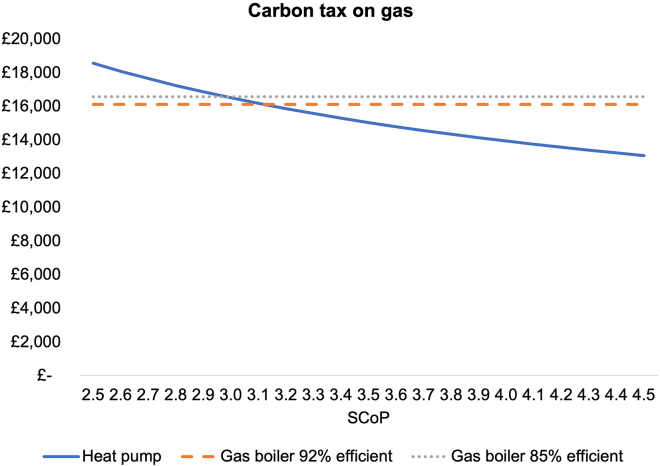
TCO under carbon tax scenario, for range of values of SCOPs To calculate the impact of a carbon tax on gas, a carbon price of £50 per kWh has been assumed, which is similar to the current carbon price in the UK Emissions Trading Scheme.^34^

Finally, the impact of a carbon tax on gas of £50/t CO_2_ is shown in Figure 4.

A carbon tax at this level would reduce the required SCOP to reach TCO parity from 3.3 to 3.0 but it would not be sufficient to achieve TCO parity for the median SCOP of 2.9 under recent UK trials.

### (Smart) Time-of-use pricing and heat pump tariffs

In addition to selected policy scenarios, we provide the TCO results for two different electricity tariffs accessible to heat pump owners as described earlier.

**Figure 5:**
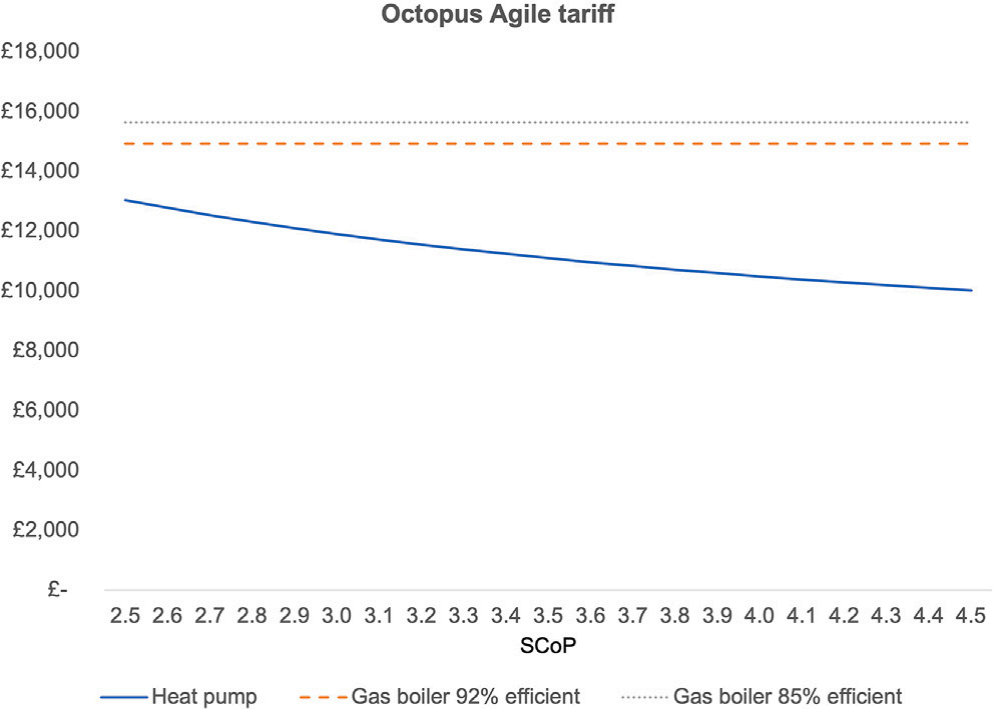
TCO with Octopus Agile tariff The same inputs as for Figure 1 were used except for electricity prices where average Octopus Agile tariffs in the period 01 January–31 March 2024 were used.^35^

At the time of writing, a time-varying tariff like Octopus Agile provides 45% average electricity cost reductions for customers (based on data shared with the authors by Octopus Energy as well as personal experience with the Agile Tariff over more than 4 years by one of the authors) and will result in lower TCO for any heat pump with an SCOP above 1.9 (Figure 5).

**Figure 6:**
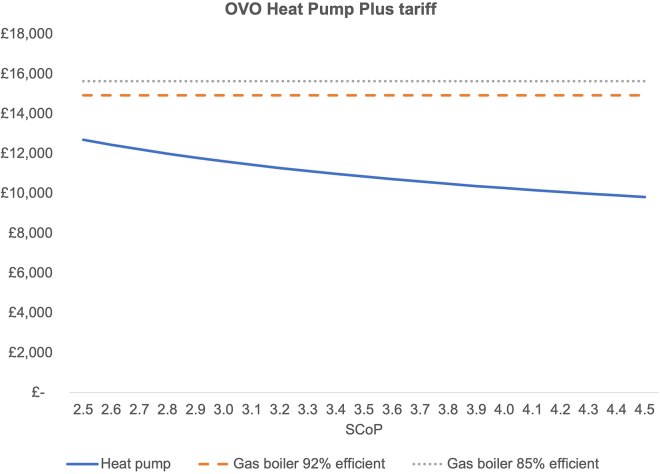
TCO with OVO Energy tariff The same inputs as for Figure 1 were used except for electricity prices where the OVO heat pump tariff was used.^36^

Using the heat pump tariff from OVO Energy, a heat pump with an SCOP larger than 1.8 will achieve lower TCO than an average gas boiler (Figure 6).

### Summary of results

**Figure 7:**
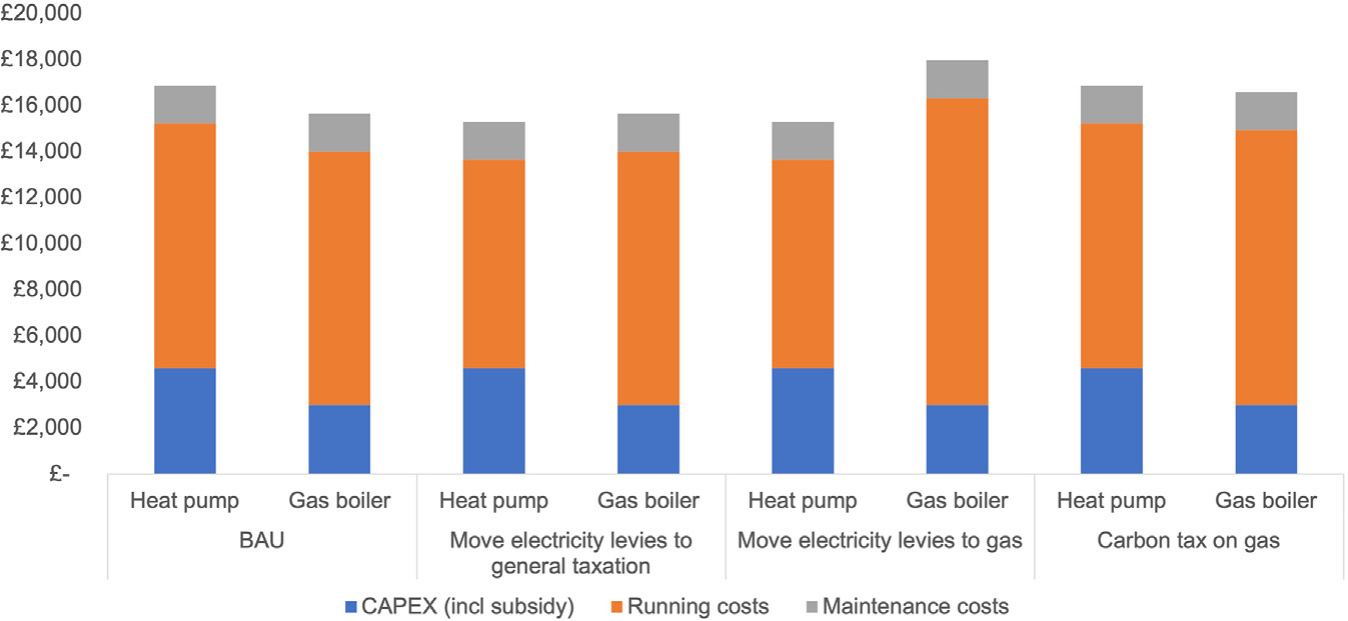
Comparison of TCO of different scenarios Data presented in figure are based on a comparison of the TCO for a heat pump with an SCOP of 2.9 (median in Electrification of Heat Trial) and an 85% efficient gas boiler for all scenarios analyzed above.

Figure 7 depicts a comparison of the TCO for a heat pump with an SCOP of 2.9 (median in Electrification of Heat Trial) and an 85% efficient gas boiler for all policy scenarios analyzed earlier.

Evidently the greatest impact on the TCO of heat pumps of the scenarios modeled is achieved by moving levies from electricity to gas bills, the second greatest by shifting levies from electricity bills to the public budget, and the third greatest by applying a carbon tax of £50/t CO_2_ on gas used for heating.

**Figure 8:**
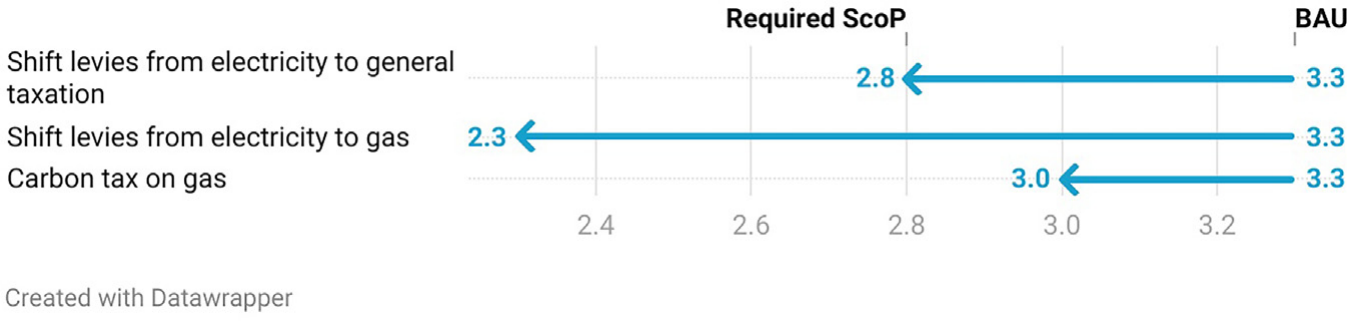
Required SCOP in order for a heat pump to have lower TCO compared to an 85% efficient gas boiler under different scenarios Input data as stated in Figures 1, 2, 3, and 4. SCOP stated reflects the first digit at which a heat pump achieves a lower TCO than a gas boiler with 85% efficiency.

Across each of the scenarios modeled, a key metric of interest is the SCOP needed for a heat pump to achieve a lower TCO than an 85% efficient gas boiler. Figure 8 gives this value for each of the scenarios. It allows for a comparison of average SCOPs from recent trials (2.9) against the different scenarios.

For those households willing to switch to either a time-varying tariff or a heat pump tariff, TCO today are positive compared to an average gas boiler though running costs can vary significantly by how the heat pump is run and variable rates are not guaranteed to be low.

**Figure 9:**
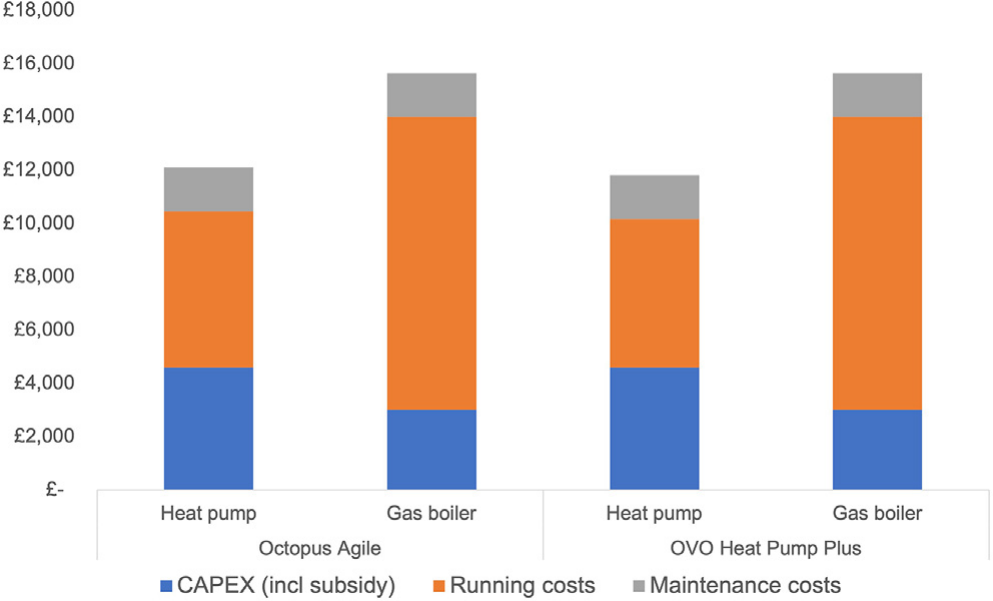
Comparison of TCO of existing dynamic ToU and heat pump specific tariffs Data presented in figure are based on a comparison of the TCO for a heat pump with an SCOP of 2.9 (median in Electrification of Heat Trial) and an 85% efficient gas boiler for all dynamic ToU and heat pump specific tariffs analyzed above.

Figure 9 depicts a comparison of the TCO for a heat pump with an SCOP of 2.9 (median in Electrification of Heat Trial) and an 85% efficient gas boiler for both a time-varying tariff or a heat pump tariff.

Compared to a gas boiler with an average efficiency of 85%, a heat pump with an SCOP of 2.9 operated on a dynamic ToU tariff (averaging 45% savings against a standard flat rate tariff), TCO is reduced by 19%. If a specific heat pump tariff offering savings of 48% per electricity unit is being used, the TCO of a heat pump is 21% lower than that of an average gas boiler. The cost parity SCoP is 1.8 and 1.9 for the OVO tariff and the Octopus Agile tariff, respectively.

### Sensitivity analysis

To ascertain the impact of our assumptions, we carry out a sensitivity analysis, changing the parameters as following.

(1) doubling the discount rate;
(2) assuming a 10% higher heat demand for buildings heated with heat pumps;
(3) assuming a 20-year lifetime for a heat pump accounting only for ¾ of the capital costs over a 15-year period; and
(4) assuming no BUS grant and a 25% heat pump cost reduction.

In the methodology section, we described why a discount rate greater than 3.5% may apply to the household decisions to adopt heat pumps. This logic justifies including sensitivity analysis for a 7.0% discount rate.

UK government analysis and recent research suggests that heating energy demand following a heat pump installation may increase by 5%–10%^37^^,^^38^ due to higher setback temperatures during off periods (e.g., during the night).

We assumed a 15-year lifetime for both heat pumps and gas boilers. However, UK government analysis suggests that for heat pumps lifetime could be as long as 20 years,^37^ which we consider in the sensitivity analysis by only accounting for ¾ of the capital costs (15/20).

Table 2 presents the TCO for the central scenario adopted for this paper and explained in detail above plus for a higher discount rate, higher heat demand for a heat pump, and a longer lifetime of a heat pump.

**Table 2:** Sensitivity analysis, giving the TCO for different scenarios (gas boiler at 85% efficiency, heat pump with SCOP of 2.9)

	Central scenario	7% discount rate	10% higher heat demand for heat pump	No BUS grant and 25% cost reduction	20 years lifetime heat pump
BAU—gas boiler	£15,640	£13,495	£15,640	£15,640	£15,640
BAU—heat pump	£16,861	£14,877	£17,924	£21,339	£15,714
Shift levies from electricity to general taxation—heat pump	£15,287	£13,594	£16,193	£19,765	£14,140
Shift levies from electricity to gas—gas boiler	£17,958	£15,327	£17,958	£17,958	£17,958
Carbon tax on gas—gas boiler	£16,572	£14,175	£16,572	£16,572	£16,572

**Figure 10:**
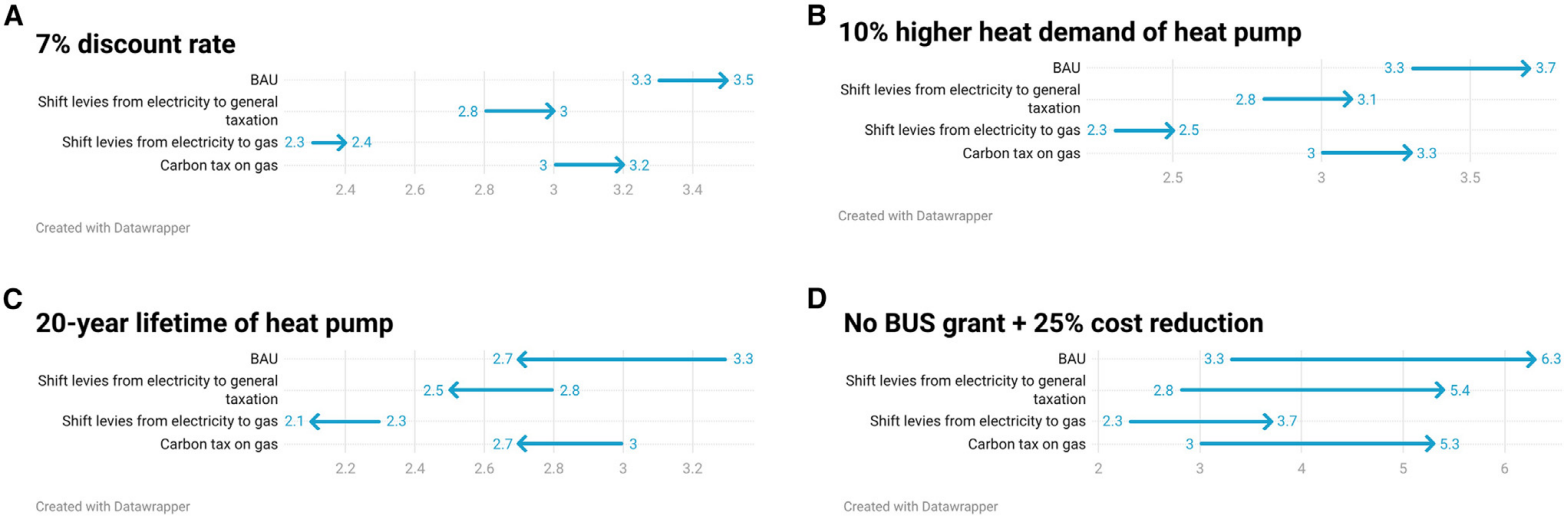
Sensitivity analysis of the required SCOP to achieve cost parity against a gas boiler (85% efficiency) Varied input data as follows: (A) changed discount rate from 3.5% to 7%; (B) assumed 10% higher heat demand for heat pumps; (C) assumed 20 years lifetime for heat pump; (D) assumed no BUS grant and 25% heat pump cost reduction.

The results of the sensitivity analysis (Figure 10) show that changing the input factors in the way described makes a significant difference to the TCO. A higher discount rate and a higher heat pump heating demand result in higher TCO for heat pumps compared to gas boilers and a longer lifetime in lower TCO for heat pumps.

A higher discount rate slightly increases the required SCOP to reach TCO parity under most scenarios. If heating demand after a heat pump install is higher, this will require higher SCOPs in return to reach TCO parity. If we assume a longer lifetime of heat pumps even under BAU, a heat pump with an SCOP above 2.7 has a lower TCO compared to gas boiler.

Importantly, if the BUS grant was removed, heat pumps could not achieve the same TCO as a gas boiler as an SCOP of more than 5 would be required under all scenarios. However, if a 25% heat pump cost reduction was achieved, which has been deemed realistic by a recent meta-study^39^ on heat pump costs, and the BUS grant was removed, it would be possible to achieve cost parity in a scenario where levies are shifted from electricity to gas with an SCOP of 3.7 and above.

Table 3 presents the sensitivity analysis carried out for ToU tariff and the heat pump tariff analysed in this paper.

**Table 3:** Sensitivity analysis, giving the TCO for different scenarios (gas boiler at 85% efficiency, heat pump with SCOP of 2.9)

	Central scenario	7% discount rate	10% higher heat demand for heat pump	No BUS grant and 25% cost reduction	20 years lifetime heat pump
BAU—gas boiler	£15,640	£13,495	£15,640	£15,640	£15,640
BAU—heat pump	£16,861	£14,877	£17,924	£21,339	£15,714
Dynamic ToU tariff—heat pump	£14,522	£10,870	£15,352	£16,577	£10,952
Heat pump specific tariff—heat pump	£12,189	£10,619	£12,354	£16,280	£10,655

A higher discount rate and a higher heat pump heating demand result in higher TCO for heat pumps compared to gas boilers and a longer lifetime in lower TCO for heat pumps.

A higher discount has almost no effect on the required SCOP to achieve cost parity with gas boilers both for the Octopus Agile and the OVO tariff. If heating demand after a heat pump install is higher, this will require a slightly higher SCOP to reach cost parity (1.9–2.1 for Octopus Agile and 1.8–1.9 for OVO). A longer lifetime of the heat pump reduces the required SCOP to 1.7 and 1.6 for Octopus Agile and OVO, respectively. If the BUS grant was removed and installation costs were to fall by 25%, heat pumps with SCOPs of 3.5 and 3.3 using Octopus Agile and OVO tariffs, respectively, are sufficient to achieve cost parity.

## Discussion

Under the BAU scenario with the median SCOP in the Electrification of Heat Trial, the TCO are higher than for an 85% efficient gas boiler. This suggests there is need for policy reform unless median efficiencies can be brought up closer to an SCOP of 3.5 in the near future.

Although the impact on the TCO would be fairly modest, removing levies from electricity bills and paying for them via general taxation would result in a situation where an SCOP of 2.8 would suffice to break even with an 85% gas boiler, meaning typical building owners would see cost savings. This policy could be applied to all domestic electricity consumption or it could be applied to electricity supplied to heat pumps only. The latter approach closely follows the example of Denmark, where only the minimum tax rate allowable under EU law applies to heat pump electricity use up to a certain limit.^12^ This approach may be more politically feasible because it reduces the need to increase general taxation. However, it would also reduce any co-benefits attained through incentivizing other forms of electrified heating. Moving levies from electricity to gas would result in a more favorable electricity to gas price ratio and 15% lower TCO for a heat pump with a 2.9 SCOP, thus providing a significant cost saving. This is by far the most impactful policy option in terms of improving the TCO of heat pumps.

This paper focused solely on the economics of heat pumps applying the approach of TCO, which narrowly looks at financial costs to the homeowner who installs a new heating system. When modeling costs for the entire building stock or energy systems as a whole, a much wider range of costs and benefits such as grid infrastructure costs would need to be taken into account for a more comprehensive analysis.^40^

Financial considerations are of course an important factor when homeowners make decisions on what heating system they decide to install. In addition, there are many other non-financial factors such as familiarity with the technology, perceived disruption, trust of installers, internal and external space for the heat pump, and (where needed) a hot water tank. These factors are not part of the analysis carried out in this paper but it is clear that they do affect the decisions made by homeowners. Public policy will need to address all of these issues holistically in order to maximize heat pump uptake (Barnes et al., 2023; Lowes et al., 2021, 2020; Rosenow et al., 2022; Rosenow and Lowes, 2020) but reducing the relative costs of heat pumps is in any case vital.

Conversely, enacting any of the policy scenarios modeled earlier has the potential to result in a wider range of impacts than changes to TCO alone. To evaluate whether to enact any of these changes, policymakers will therefore need to consider these broader implications, including the impact of any reforms on energy efficiency, the public budget, and equity.

In our calculations, we assumed grant support for heat pump installs at the current BUS levels. Governments may decide to reduce the level of support in the future once the costs of heat pump installs fall. Recent research on heat pumps indicates that significant cost reductions of up to 25% could be achieved in particular through cost savings in the installation process.^39^ To what extent future cost reductions can be realized remains to be seen but recent investment by major companies in the UK heat pump sector could pave the way to a lowering of the capital costs of heat pumps (Barnes et al., 2023).

For policy makers working to decarbonize the building sector, we suggest that the following considerations should be core to energy policies:

Upfront grants may form an important part of the policy package, managing retrofit cost impacts and supporting positive relative heat pump lifetime costs.
Levies can be shifted to rebalance prices and support heat pump adoption. Upward pressure on electricity bills should be avoided.
Upward pressure on fossil fuel heating via carbon taxation can also drive positive heat pump economics but issues of equity need to be managed.
Efforts should be taken to reduce upfront heat pump costs and increase heat pump performance though managing both simultaneously may be difficult.
Heat pump operating efficiency is a key determinant of the TCO. Policy makers are well advised to ensure that heat pumps operate at high SCOPs to maximize cost savings.

Overall, the economics of heat pumps can be supported through the utilization of traditional policy tools but efforts to encourage innovation in installation and performance can also drive cost reductions. Flexible pricing can also have a very positive impact on heat pump economics. Efforts to reduce relative heat pump costs will likely need to come alongside efforts to reduce wider social and political barriers.

Our analysis offers insights that are complementary to other techno-economic cost studies on heating carried out elsewhere. Future work could segment housing types and further refine the sensitivity analysis by looking at a wider range of variations. This will require a better and more granular collection of input data currently not available.

### Limitations of the study

This analysis uses averages in the calculations including the average heat demand of a building, average electricity and gas prices, average installation costs, and average efficiencies. We also did not model different load profiles of households in the analysis of ToU tariffs. In reality, all of these factors will differ building by building. The sensitivity analysis we carried out addresses some of those limitations but not all of them. Future research could provide more granularity of different housing architypes and expand the sensitivity analysis based on a wider range of input parameters.

## Resource availability

### Lead contact

Further information and requests for resources and reagents should be directed to and will be fulfilled by the lead contact, Jan Rosenow (jan.rosenow@ouce.ox.ac.uk).

### Materials availability

This study did not generate new unique materials.

### Data and code availability

• Data: the source data are available as listed in the key resources table.
• Code: no code was generated for the analysis. The Excel file with all calculations has been deposited at Mendeley at https://doi.org/10.17632/xdzxmt2zg6.1 and is publicly available as of the date of publication.
• All other items: any additional information required to reanalyze the data reported in this paper is available from the lead contact upon request.

## Acknowledgments

Jacob Barnes received funding from the Centre for Research into Energy Demand Solutions (grant reference number EP/R 035288/1).

## Author contributions

Conceptualization, J.R; methodology, J.R. and S.O.; investigation, J.R. and S.O.; writing—original draft, J.R., J.B., S.O., R.G. and R.L.; writing—review and editing, J.R., J.B., R.G., and R.L.; supervision, J.R.

## Declaration of interests

The authors declare no competing interests.

## STAR★Methods

### Key resources table

**Table:** 

REAGENT or RESOURCE	SOURCE	IDENTIFIER
**Deposited data**		

Average cost of installing a heat pump	MCS^22^	https://datadashboard.mcscertified.com/InstallationInsights
Average cost of installing a gas boiler	HM Government^23^	https://www.gov.uk/government/news/heat-pump-grants-increased-by-50-per-cent
Average gas demand (used to derive average heat demand)	Department for Energy Security and Net Zero^24^	https://www.gov.uk/government/statistics/national-energy-efficiency-data-framework-need-consumption-data-tables-2023
Average gas demand for cooking only (used to derive average heat demand)	Department for Business, Energy & Industrial Strategy^25^	https://www.gov.uk/government/statistics/energy-consumption-in-the-uk-2021
Average gas boiler efficiency	Energy Saving Trust^26^	https://www.gov.uk/government/publications/in-situ-monitoring-of-efficiencies-of-condensing-boilers-and-use-of-secondary-heating-trial-final-report-2009
Average heat pump efficiency	Energy Systems Catapult^29^	https://es.catapult.org.uk/report/electrification-of-heat-interim-heat-pump-performance-data-analysis-report/
Electricity and gas price forecasts	DESNZ.^27^	https://www.gov.uk/government/publications/valuation-of-energy-use-and-greenhouse-gas-emissions-for-appraisal
Discount rate	HM Treasury^28^	https://www.gov.uk/government/publications/the-green-book-appraisal-and-evaluation-in-central-government
Default electricity and gas prices	34. Ofgem^41^	https://www.ofgem.gov.uk/energy-price-cap
Octopus Agile tariffs	Octopus Energy^35^	https://agile.octopushome.net/historical-data
OVO heat pump tariff	O OVO^36^	https://www.ovoenergy.com/heat-pump-plus
Levies	Ofgem^42^	https://www.ofgem.gov.uk/energy-policy-and-regulation/policy-and-regulatory-programmes/energy-price-cap-default-tariff-policy/energy-price-cap-default-tariff-levels

### Experimental model and study participant details

The study included no experiments or study participants.

### Method details

In order to determine the total costs of ownership (TCO) of heat pumps and gas boilers in the scenarios outlined, a number of calculations need to be performed using a range of input parameters described in this section.

#### TCO calculation

The TCO for heat pumps is calculated as follows:

(Equation 1) $$TCO=C_{CAPEX}-C_{BUS}+C_{OPEX,T}$$

where *C*_*CAPEX*_ is the cost of the installation on the heating system [£], *C*_*BUS*_ is the subsidy payment for heat pumps through the Boiler Upgrade Scheme [£], *C*_*OPEX,T*_ is the net present value (NPV) running costs in period *T*.

*C*_*OPEX*_*,*_*T*_ is given by:

(Equation 2) $$C_{OPEX,T}=A\cdot \frac{\left[1+i-{\left(\frac{1}{\left(1+i\right)}\right)}^{T-1}\right]}{i}$$

where *A* is the annual running cost including energy costs and maintenance. *T* is the assumed lifetime of the heating system [years] and *i* is the discount rate [%]. We discount from the second year of operation.

*A* for heat pumps is given by:

(Equation 3) $$A=H*\frac{1}{E_{SCOP}}*F+M$$

where *H* is the heat demand [kWh/year], *E*_*SCOP*_ is the seasonal coefficient of performance of the heat pump [SCOP], *F* is the energy price [£/kWh], and *M* is the maintenance cost in a given year [£/year].

Hence Equation 2 assumes that all these annual costs are discounted by the same discount rate.

The UK’s Green Book used for policy appraisal recommends not to account for inflation in future cost projections stating that “the best practice approach is to first convert costs or benefits to a real price basis, and then perform the discounting adjustment”.^28^

For gas boilers the calculation is:

(Equation 4) $$TCOg=C_{CAPEXg}+C_{OPEXg,T}$$

The form of the equation is the same as for Equation 1 but the subscript *g* indicates that this is for gas. The *C*_*OPEXg*_ for gas boilers in a given year is given by:

(Equation 5) $$C_{OPEXg}=H*\frac{1}{E_{Eff}}*F_{g}+M_{g}+S_{g}$$

where *H* is the heat demand [kWh/year], *E*_*Eff*_ is the efficiency of the gas boiler [%], *F*_*g*_ is the energy price [£/kWh], *M*_*g*_ is the maintenance cost in a given year and *S*_*g*_ is the standing charge [£/year].

Again we use Equation 2 to include the effects of the discount rate, again applied from the second year of operation.

#### Heat demand

The heat demand (*H*) for a typical household can be estimated at approximately 9,962 kWh per year, taking into account a boiler efficiency (*E*_*Eff*_) of 85% (as detailed below), and based on the median annual gas consumption of 12,020 kWh,^24^ with an exclusion of the 2.5% of gas allocated for cooking.^25^

#### Gas boiler efficiency

Gas boiler efficiency (*E*_*Eff*_) is considered in the calculation above. The minimum standard for new gas boilers, as stipulated by the Energy-related Products Regulations^43^ is set at 92% efficiency.

However, the actual efficiency of a gas boiler can vary considerably and hinges on whether the flow temperature, which denotes the temperature of the water circulating through the house's radiators, is low enough for the boiler to condense; it's worth noting that most boilers do not achieve condensation.^44^

In the UK, boilers are typically oversized,^45^ which has an impact on their efficiency. Furthermore, the presence and adequacy of controls for the heating system also affect its overall efficiency.

There is limited peer-reviewed literature on in-situ boiler efficiencies.^45^^,^^46^^,^^47^ Detailed on-site monitoring studies of condensing boilers commissioned by the UK government revealed an average measured efficiency of 82.5% for combination boilers and 80.3% for heating-only boilers.^26^ This does not seem to include circulation losses. The study concluded that “the in-situ performance of the boilers is significantly less than the rated […] seasonal efficiency”.^26^ Consequently, it is more realistic to assume that, in most cases, the 92% efficiency standard is not being met. However, the last comprehensive monitoring study is now quite dated and boiler efficiency standards have become more stringent. It may be possible that higher efficiencies are reached if the monitoring study was repeated today. Therefore, for the calculations using boiler efficiency, both a 92% boiler efficiency and a lower 85% efficiency are considered.

#### Heat pump efficiency

To calculate heat pump running costs we assess a range of heat pump efficiencies (*E*_*SCOP*_). A heat pump's actual efficiency is contingent upon the achieved coefficient of performance (COP). Achieving three or more units of heat for every unit of electricity consumed is feasible due to the heat pump's capacity to extract thermal energy from the surrounding air, ground, or water.^48^ Given that the system's performance fluctuates throughout the year, it is advisable to employ the seasonal coefficient of performance (SCOP) when conducting cost analyses^49^ representing the ratio of the amount of heat delivered compared to the electricity input to run a heat pump over the course of a year. Much like boilers, the efficiency of a heat pump is subject to variation and depends on various factors. One crucial parameter is once again the flow temperature: a narrower temperature differential between the heat source and flow temperature leads to a higher coefficient of performance (COP).^38^

Previous field studies in the UK have shown that poor design and installation sometimes resulted in lower efficiencies.^50^^,^^51^ However, this can be avoided by appropriate design, installation and maintenance of the heat pump. More recent studies of achieved SCOPs in the UK as part of the Electrification of Heat Trial indicate that the median SCOP was 2.94 (excluding circulation pumps). However, even for air source heat pumps SCOPs of 4.0 have been demonstrated in the UK subject to the right training of the installer and high-quality models.^21^ Because SCOPs vary significantly by installation we perform the TCO calculations under different scenarios for a range of SCOPs.

#### Fuel price

A variety of fuel prices (*F*) are used in our calculations including standard domestic prices. Base price forecasts for future years were taken from DESNZ’s Green Book Assumptions.^27^ These forecasts were developed according to long run trends in fuel prices. It was therefore assumed that these forecasts did not account for future changes in levy costs on bills. Therefore, the price forecasts were adjusted according to changes in levy costs per unit of energy from the 2023 baseline. For example, if levies on electricity bills decreased by 0.1£/kWh, then an adjustment of -0.1£/kWh was made to the retail price forecast.

#### Standing charge

Both electricity and gas services come with a standing charge (*S*), a fixed amount payable per day. However, households heated with gas always incur the standing charge for electricity, whereas homes without a gas connection and relying solely on electric heating do not have a gas standing charge. As a result, it is reasonable to disregard the standing charge for electricity, as both gas boiler-heated homes and those with heat pumps are obligated to pay it, irrespective of their heating system. In the case of gas, the energy price cap set by Ofgem data determines a standing charge of £0.30 per day.^41^

Future standing charges were estimated according to the average historical ratio of unit charges to standing charges from the Ofgem price cap methodology.^20^ Note that our analysis does not take into account potential future changes to standing charges for electricity which would potentially also be impacted by electricity system costs related to the additional load from heat pumps.

#### Capital costs

The capital cost (*C*_*CAPEX*_) of an installation includes both the equipment and the labour involved in the installation. The average cost of an air source heat pump install (including the heating system and labour) in 2022 was £12,088 according to Microgeneration Certification Scheme (MCS) dashboard.^22^ A new gas combination boiler install (including the heating system and labour) costs on average £3,000.^37^

#### Discount rate

Which discount rate to use has been subject to intense debate in the literature on households and behaviour regarding energy technology adoption. Of critical importance is whether discount rates relate to consumer decisions or social cost-benefit analysis.^52^^,^^53^

Hamamoto^54^ recently conducted an assessment of consumers' discount rates related to energy-saving investments, utilising hedonic pricing and qualitative choice models. The identified rates ranged from 10.7% to 13.6%, with the possibility of reaching as high as 50%. In comparison, Fujita^55^ estimates discount rates for commercial buildings at 8%-10% for comparison.

Social discount rates are typically lower than individual discount rates. Given that our analysis is about policy options rather than modelling individual consumer decisions we use a social discount rate. In line with the UK Government’s Green Book we use a discount rate (*i*) of 3.5%.^56^ Discount rates may well be higher but the Green Book guidance suggests not adjusting for inflation in calculating prices of for future years.

For the purpose of this paper we also carry out sensitivity analysis and double the discount rate from 3.5% to 7.0%.

#### Levies

Domestic electricity bills are composed of many different elements. Both electricity and gas are taxed at 5% value-added tax in the UK. Additional policy costs (called levies) are applied to electricity and gas (Table 1). The share of levies of electricity and gas prices paid by domestic customers varies as prices a volatile. In Q3 2024 levies for electricity were 17% of electricity bills and levies for gas were set at 6%.^42^

Some of those levies are for ongoing policies and programmes whereas others are legacy policy cost. The policies covered by levies include support programmes for renewable energy, energy efficiency and energy bill rebates for low-income customers.

The following levies have been taken into account in the analysis:

- Renewables Obligation: This scheme was established to promote the generation of electricity from eligible renewable sources within the United Kingdom. The scheme was initiated in Great Britain in 2002, followed by its implementation in Northern Ireland in 2005. Under the scheme, electricity suppliers were annually obligated to provide a specified number of Renewables Obligation Certificates per MWh of electricity they supplied to their customers during each obligation period.
- Feed-in Tariffs: The Feed-in Tariffs scheme was created by the government to encourage the adoption of renewable and low-carbon electricity generation. Initiated on April 1, 2010, this scheme mandated that licensed electricity suppliers who participated in it must make payments for the electricity produced and exported by accredited installations.
- Energy Company Obligation: The Energy Company Obligation is a government energy efficiency programme in Great Britain aimed at addressing fuel poverty and contributing to the reduction of carbon emissions. The scheme originated in 1994 and places obligations on energy suppliers to fund the installations of energy saving measures.
- Warm Home Discount: The Warm Home Discount scheme is designed to assist individuals with low incomes who are vulnerable to cold-related illnesses or are predominantly living in fuel poverty. It was implemented in April 2011 and has been extended by the government through March 2026. This scheme mandates that domestic energy suppliers serving over 1000 customers must offer an annual discount on customer bills.
- Contracts for Difference (CfD): The CfD scheme serves as the primary government mechanism for endorsing low-carbon electricity generation. In this framework, if the market price for electricity produced by a CfD Generator (the reference price) falls below the predetermined Strike Price outlined in the contract, the Low Carbon Contracts Company makes payments to the CfD generator to compensate for the shortfall. Conversely, when the reference price exceeds the Strike Price, the Low Carbon Contracts Company receives payments from the CfD generator.

The energy regulator Ofgem^20^ provides the cost these levies put on electricity and gas bills in £/kWh except for the Warm Home Discount which is charged per customer.

To calculate future levies in £/kWh the following assumptions have been made:

To estimate the future energy levies on consumer bills, first the current levy structure was analysed. Data on the current levies placed onto domestic consumer bills was found in the relevant Ofgem workbooks^20^ used for calculating fuel prices under the price cap.

Complementary data on the current and historic consumption of natural gas and electricity by end use was taken from the Department of Energy Security and Net Zero’s annual Energy Trends data.^30^^,^^31^ This allows for the estimation of current funding pots for relevant schemes funded via levies on domestic consumer bills and is a useful cross reference for future levy forecasts.

For each individual scheme funded via levies on consumer bills, a different approach was taken to forecasting the total funding requirement over time. Where appropriate, total funding requirements were scaled down according to projections in future energy usage^32^ and the share of non-domestic consumption, which is exempt from levy costs, for example, through the Energy Intensive Industries Exemption.^57^^,^^58^ The total funding requirements were then mutualised across the total eligible energy consumption or total eligible customer base according to the current funding structure and data from the National Grid ESO’s Consumer Transformation scenario^33^ on future energy consumption. For example, the Energy Company Obligation is funded via a levy per unit of energy consumed and charges are split equally between gas and electricity consumers, so levies were apportioned accordingly. This was used to forecast levy charges per customer and per unit of energy consumed. More detail on forecast methodologies for each relevant levy cost can be found in Table 1.

For the purpose of this paper all levies have been recalculated in £/kWh using the median electricity consumption.^24^

In the policy scenario of moving levies to the public budget, levies are subtracted from the fuel price (*F*):

*C*_*OPEX*_ for heat pumps in a given year is calculated as follows:

(Equation 6) $$C_{OPEX}=H*\frac{1}{E_{SCOP}}*\left(F-L_{elec}\right)+M$$

*L*_*elec*_ is the cost of levies on electricity (£/kWh) in a given year forecasted using the methodology above.

In the scenario of shifting levies from electricity to gas the total amount of social and environmental levies of electricity in a given year is added to gas prices based on total domestic gas consumption.

Hence *C*_*OPEX*_ for gas boilers in a given year is calculated as follows:

(Equation 7) $$C_{OPEXg}=H*\frac{1}{E_{Eff}}*\left(F_{g}+L_{elec}\right)+M_{g}{+S}_{g}$$

In reality, policy makers may of course chose to only shift certain types or parts of levies from electricity to gas.

#### Carbon tax

To calculate the impact of a carbon tax on gas a carbon price of £50 per kWh has been assumed which is similar to the current carbon price in the UK Emissions Trading Scheme.^34^

*C*_*OPEX*_ for gas boilers in a given year is calculated as follows:

(Equation 8) $$C_{OPEX}=H*\frac{1}{E_{Eff}}*\left(F_{g}+T_{n}\right)+M_{g}+S_{g}$$

*T*_*n*_ is the assumed carbon tax calculated in a given year in £/kWh.

### Quantification and statistical analysis

No statistical analysis was performed as part of this paper.
